# The effects of intranasal esketamine on on-road driving performance in patients with major depressive disorder or persistent depressive disorder

**DOI:** 10.1177/02698811221078764

**Published:** 2022-02-25

**Authors:** Francis M Dijkstra, Aurora JAE van de Loo, Smedra Abdulahad, Else R Bosma, Mitch Hartog, Hendrikje Huls, Dianne C Kuijper, Esther de Vries, Bhavna Solanki, Jaskaran Singh, Leah Aluisio, Peter Zannikos, Frederik E Stuurman, Gabriël E Jacobs, Joris C Verster

**Affiliations:** 1 Centre for Human Drug Research (CHDR), Leiden, The Netherlands; 2 Department of Psychiatry, Leiden University Medical Centre (LUMC), Leiden, The Netherlands; 3 Institute for Risk Assessment Sciences (IRAS), Utrecht University, Utrecht, The Netherlands; 4 Utrecht Institute for Pharmaceutical Sciences (UIPS), Utrecht University, Utrecht, The Netherlands; 5 Janssen Research & Development, La Jolla, CA, USA; 6 Janssen Research & Development, LLC, Raritan, NJ, USA; 7 Centre for Human Psychopharmacology, Swinburne University, Melbourne, Australia

**Keywords:** Major depressive disorder, persistent depressive disorder, esketamine nasal spray, driving, SDLP

## Abstract

**Background:**

Intranasal esketamine demonstrates rapid improvement of depressive symptoms. However, transient adverse effects (dissociation, sedation and dizziness) may occur, which could impact driving performance.

**Aims:**

To evaluate the effects of 84 mg intranasal esketamine on driving performance in unipolar major depressive disorder (MDD) or persistent depressive disorder (PDD) patients.

**Methods:**

The study consisted of two parts. Part A was a single-blind, double-dummy, randomized three-period, cross-over study to compare effects of esketamine versus placebo on next morning driving, 18 ± 2 h post-treatment. Alcohol was administered to demonstrate assay sensitivity. In Part B, same-day driving, 6 ± 0.5 hours post-treatment, was assessed during twice weekly esketamine administration for 3 weeks. Twenty-seven patients with mild-to-moderate MDD or PDD without psychotic features completed a 100 km on-the-road driving test on a public highway in normal traffic. The primary outcome was standard deviation of lateral position (SDLP; cm; weaving of car).

**Results:**

In Part A, alcohol impaired driving performance compared to placebo: Least-square means (95% CI), *p*-value for delta SDLP (cm) compared with placebo: (ΔSDLP = + 1.83 (1.03; 2.62), *p* < 0.001), whereas esketamine did not: (ΔSDLP = −0.23 (−1.04; 0.58), *p* = 0.572). In Part B, weekly driving tests showed no differences between placebo baseline SDLP and after esketamine administration over 3 weeks: Day 11: (ΔSDLP = −0.96 (−3.72; 1.81), *p* = 0.493), Day 18: (ΔSDLP = −0.56 (−3.33; 2.20), *p* = 0.686) and Day 25: (ΔSDLP = −1.05 (−3.82; 1.71), *p* = 0.451).

**Conclusions:**

In this study, esketamine did not impair on-road driving performance the next morning following a single dose, or on same day after repeated administration.

## Introduction

Major depressive disorder (MDD) is a commonly occurring neuropsychiatric condition which affected over 264 million people worldwide in 2017 (James et al., 2018) and is associated with excess mortality with an estimated median years of potential life loss of 10 years (Vos et al., 2012; Walker et al., 2015). Although several evidence-based pharmacological treatments for MDD are available, up to 50–60% of patients suffer from treatment-resistant depression (TRD) (Fava, 2003). These patients do not benefit from available antidepressant medications despite an adequate therapeutic dose and duration of therapy (Fava, 2003). Moreover, most registered antidepressant drugs modulate central monoaminergic neurocircuits and typically are effective after only 4–7 weeks (Murrough and Charney, 2012). Together, this illustrates the need to develop antidepressant drugs with novel mechanisms of action and improved efficacy profiles that attain rapid symptomatic relief.

In 2019, nasal esketamine was approved by the United States Food and Drug Administration and other health authorities worldwide to be used in conjunction with an oral antidepressant for the treatment of TRD in adults (SPRAVATO, 2020). Compared to existing antidepressants esketamine has a different mechanism of action and onset of antidepressant effects occurs as early as 2 h after administration (Daly et al., 2018; Singh et al., 2016). In the United States, nasal esketamine is also approved for the treatment of depressive symptoms in adults with MDD with acute suicidal ideation or behaviour (SPRAVATO, 2020). In the European Union, nasal esketamine together with an oral antidepressant is approved as acute short-term treatment, for the rapid reduction of depressive symptoms in adults with a moderate to severe episode of MDD, which according to clinical judgement constitute a psychiatric emergency (SPRAVATO SmPC).

The pharmacokinetics of nasally administered esketamine have been characterized and summarized in product labelling (SPRAVATO, 2020, SPRAVATO SmPC). The maximum plasma concentration (*C*_max_) of esketamine following intranasal administration is reached within 20–40 min. After reaching *C*_max_, there is a biphasic decline in plasma concentration for the first 2–4 h and a mean terminal half-life (*t*_1/2_) ranging from 7 to 12 h. Esketamine is extensively metabolized by the cytochrome P450 enzymes, mainly 3A4 and 2B6. N-demethylation of esketamine to form noresketamine is the major metabolic pathway. Noresketamine has a similar elimination profile with a mean *t*_1/2_ of approximately 8 h.

Intranasal esketamine is generally well tolerated, but adverse reactions of dissociation, dizziness, nausea, sedation and vertigo commonly occur after dosing (SPRAVATO, 2020). Furthermore, ketamine, the racemic mixture of arketamine and esketamine, is a medicinal product that has been reported to be abused (SPRAVATO, 2020). Withdrawal symptoms of cravings, anxiety, shaking, sweating and palpitations have been reported in individuals dependent on ketamine. To minimize the risk of abuse, misuse and diversion of intranasal esketamine, administration must take place under the direct supervision of a healthcare professional (SPRAVATO, 2020). The adverse events (AEs) of dissociation, dizziness, nausea, sedation and vertigo generally resolve the same day and attenuate with repeated dosing (Daly et al., 2018; Popova et al., 2019; Wajs et al., 2020). Symptoms of dissociation and sedation have been shown to resolve within 1.5–2 h (Daly et al., 2018; Popova et al., 2019; Wajs et al., 2020). However, considering the sedative and dissociative effects associated with intranasal esketamine (SPRAVATO, 2020, SPRAVATO SmPC), it was deemed crucial to investigate its effects on driving ability.

Driving is a complex activity that involves cognitive, perceptual, and motor activities. As noted in a guidance for industry that was issued by the Food and Drug Administration (FDA, 2017), collection of objective information about how a drug affects driving ability, with higher specificity than more general tests of central nervous system (CNS) function, may be necessary to enable safe use of a drug that has pronounced CNS impairing effects.

The current gold standard to measure driving performance is the on-the-road driving test (O’Hanlon et al., 1982). This test has been used to measure driving effects of various CNS active compounds in more than 75 clinical trials (Verster and Mets, 2009) . Furthermore, it has the methodological advantages of good test–retest reliability and closely mimicking real-life driving as it is performed on the public highway (Verster and Roth, 2011). The primary outcome of the on-the-road driving test is the standard deviation of lateral position (SDLP), which reflects weaving of the motor vehicle (Verster and Roth, 2011).

In a prior on-road driving study in healthy volunteers assessing same-day driving performance at 8 h after dosing, no significant difference in SDLP was found between 84 mg intranasal esketamine and placebo, while a significant effect was found for 30 mg mirtazapine (positive control) (van de Loo et al., 2017). For esketamine, driving performance was deemed to be ideally investigated in the target TRD population as these patients might differ from healthy controls with regards to comorbid disorders, use of concomitant medication and response to esketamine treatment. Next to that, depression itself increases the relative risk of becoming involved in a car accident (Vaa, 2003). Since studies in TRD are complex in terms of logistics and enrolment, a broad category of unipolar MDD or persistent depressive disorder (PDD)/dysthymia patients was recruited for the present study. Although such patients are not identical to TRD patients, they resemble the target population more closely than healthy volunteers.

Taken together, the main purpose of the present study was to evaluate the effects of esketamine at the highest therapeutic dose, 84 mg intranasally on next-day driving performance following single administration, and on same-day driving performance following repeated administration in MDD or PDD patients as measured by the on-the-road driving test. Secondary objectives of this study were to investigate the safety and efficacy of 84 mg intranasally administered esketamine.

## Methods

The study was approved by Stichting Beoordeling Ethiek Biomedisch Onderzoek (BEBO) Medical Ethics Committee and registered at clinicaltrials.gov under number NCT02919579. Written informed consent was obtained from all patients before the study start, and the study was performed according to the International Committee on Harmonization of Good Clinical Practice (ICH GCP) guidelines as laid down in the Declaration of Helsinki and its latest amendments. The study was sponsored by Janssen Research and Development and conducted in collaboration between the Centre for Human Drug Research, Leiden, The Netherlands (clinical assessments) and Utrecht University, Institute for Risk Assessment Sciences (on-road driving assessments). The study was conducted from 18 October 2016 to 4 July 2018.

### Study design

**Figure 1.**
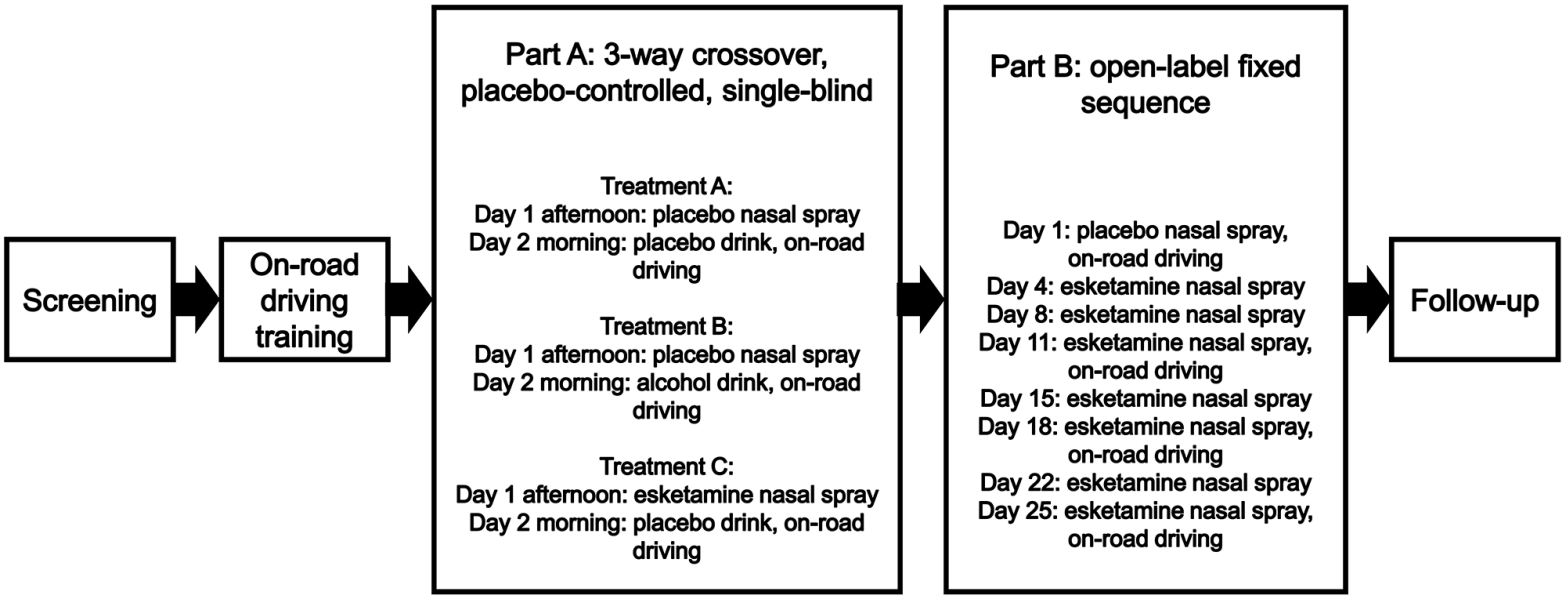
Schematic overview of study.

The study consisted of two parts (Figure 1). Part A was designed to test the effect of a single intranasal esketamine administration on next-day driving performance compared to placebo as most AEs associated with the use of intranasal esketamine are known to dissolve the same day (Daly et al., 2019; Popova et al., 2019; Wajs et al., 2020). This study part had a single-blind, double-dummy, placebo-controlled, randomized three-period and cross-over design. In line with recommendations by the FDA (2017), an alcohol-containing beverage intended to achieve a blood alcohol concentration (BAC) of 0.05% (i.e. Dutch legal driving limit) was used as the active control to demonstrate assay sensitivity. Part A consisted of three 2-day visits, during which patients received one treatment per visit in a randomized order. On the visits in which patients were randomized to receive esketamine or placebo intranasal spray, they self-administered study medication in the afternoon at 4 PM, and performed a driving test the next morning (between 8 AM and 12 PM), following a night sleep. The times for dosing and performing the driving test, resulting in a time window of testing driving performance at 18 ± 2 h after dosing, were based on pragmatic reasons as this resulted in a feasible time schedule. On the visit in which patients were randomized to receive an alcohol- or a placebo-containing beverage, patients drank the beverage approximately 45 min prior to the driving test. The driving test started immediately after it was demonstrated, based on results of two or more breathalyser assessments, that the BAC was ⩽ 0.05%. To maintain blinding of the study, patients were not informed about the outcome of the BAC readings and breathalyser tests were also performed after administration of the placebo beverage. After completion of the driving test, the BAC was measured again. The visits were separated by a wash-out period of 5–14 days.

Part B was designed to assess the effect of repeated administration of intranasal esketamine on same-day (6 ± 0.5 h after dosing) driving performance (Figure 1). This study part had an open-label, placebo-controlled, fixed-sequence design. The 6-h timepoint for the driving test was chosen as it was expected that most AEs would be resolved by then (Daly et al., 2018; Popova et al., 2019; Wajs et al., 2020). On Day 1, patients self-administered intranasal placebo spray and on Days 4, 8, 11, 15, 18, 22 and 25, the patients self-administered 84 mg esketamine intranasal spray at 9 AM. On Day 1, 11, 18 and 25 driving tests were performed between 2:30 and 3:30 PM. A time window of ± 1 day for Days 4–25 was permitted. A safety follow-up visit was performed 7–10 days after completion of Part B or when patients early withdrew. This meant that the total study duration was up to 98 days (screening period of 21 days, Part A consisting of three 2-day periods with 5–14 days wash-out between each study drug administration, 5–14 days of wash-out between Parts A and B, Part B consisting of up to 26 days and a follow-up safety visit 7–10 days after completion of Part B).

To minimize the potential side-effects of nausea and vomiting (SPRAVATO, 2020, SPRAVATO SmPC), esketamine was administered at least 2 h after food intake in both study parts and fluid consumption was restricted for at least 30 min before the first intranasal spray of study drug. Patients were instructed to refrain from blowing their nose for at least 1 h after the last intranasal spray. All patients returned to the research centre after completing the driving test and a safety assessment was performed by a physician prior to discharge.

### Study population

Males and females between the ages of 22 and 60, having a body mass index (BMI) between 18 and 32 kg/m^2^, a body weight ⩾ 50 kg and meeting the *Diagnostic and Statistical Manual of Mental Disorders* (4th ed.; DSM-IV) or *Diagnostic and Statistical Manual of Mental Disorders* (5th ed.; DSM-5) criteria of (recurrent) symptomatic MDD without psychotic features or PDD/dysthymia were included in this study. Diagnosis was based on clinical assessment, including the Mini International Neuropsychiatric Interview (MINI) and confirmation by the attending general practitioner, psychiatrist or clinical psychologist. Severity of MDD/PDD was assessed by the Montgomery Åsberg Depression Rating Scale (MADRS; Montgomery and Asberg, 1979), patients with a total score of ⩾ 18 at screening were included. Patients with a primary sleep disorder, a history of moderate or severe substance use disorder according to DSM-IV or DSM-5 within 1 year before screening were excluded. Comorbid generalized anxiety disorder, social anxiety disorder and panic disorder were allowed under condition that MDD/PDD was the primary diagnosis. Patients were not allowed to use medication that can cause sedation, such as benzodiazepines, metopimazine, scopolamine and zolpidem, or any Category III drugs and Category II antidepressants included in the categorization system of the International Council on Alcohol, Drugs and Traffic Safety (ICADTS) from 1 week prior to the first dose of study drug until completion of the last driving test (Verster and Mets, 2009). To participate in the study, patients must have had a valid driving licence in good standing for more than 60 months and must have driven regularly in the year prior to screening.

### Treatments

#### Intranasal esketamine spray and placebo spray

Esketamine intranasal (84 mg) was administered as an aqueous solution of esketamine hydrochloride (16.14% w/v; equivalent to 14% w/v of esketamine base). The nasal spray device delivered 16.14 mg esketamine hydrochloride (14 mg esketamine base) per 100 μL spray. Each device contained sufficient volume for two sprays (28 mg). Each patient self-administered the nasal spray under medical monitoring, three devices with 5 min in between each device (total dose 84 mg). Patients were trained on self-administration with a placebo device as a part of the screening procedures. The solution for the placebo intranasal spray contained a colourless solution of water for injection with a bittering agent (denatonium benzoate (Bitrex^®^) at a final concentration of 0.001 mg/mL) added to simulate the taste of the intranasal solution with active drug. Benzalkonium chloride was added as a preservative at a concentration of 0.3 mg/mL.

#### Alcohol and placebo beverage

Friel’s equation which takes into account body weight and gender was used to determine the amount of alcohol needed to reach a breath alcohol concentration, correlating with a BAC of 0.05% (Friel et al., 1999). The beverage was prepared by mixing the appropriate volume of alcohol with sugar-free orange juice up to a final volume of 250 mL and was blinded by adding a taste masker (menthae piperitae aetheroleum, Ph. Eur.). The placebo beverage (no alcohol) was also prepared with sugar-free orange juice up to a volume of 250 mL and was blinded by adding the same taste masker.

### Pharmacokinetic assessments

Esketamine and noresketamine concentrations in blood plasma samples were analysed using a validated, specific and sensitive liquid chromatography coupled to tandem mass spectrometry method. The quantification range was 0.500–500 ng/mL for both esketamine and noresketamine.

### Pharmacodynamic measures

#### On-road driving

**Figure 2.**
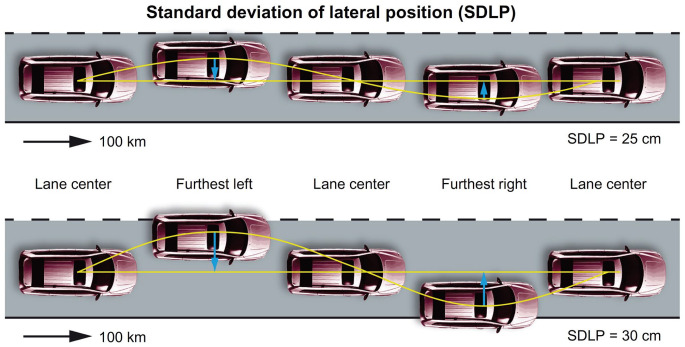
Schematic representation of SDLP.

The primary outcome of the on-the-road driving test was the SDLP (cm) (Figure 2). The secondary outcome measure of the driving test was the standard deviation of speed (SDS, km/h). Control variables were mean lateral position (MLP, cm) and mean speed (MS, km/h).

The procedures for the on-the-road driving test are described by Verster and Roth (2011). Patients performed an on-the-road training test during screening to become familiar with the test circuit and procedures. Learning effects among people who drive regularly, as selected for this study, are unlikely (Verster and Roth, 2011). Patients drove on a public highway, a 100 km track between the cities of Utrecht and Arnhem (The Netherlands) in an instrumented vehicle. The test was conducted during normal traffic to closely mimic real-life driving. Patients were instructed to drive with a steady lateral position within the right (slower) traffic lane, while maintaining a constant speed and instructed to overtake slower driving vehicles. To ensure patient safety during driving tests, their clinical and self-reported status were evaluated prior to each test and a licensed driving instructor with access to dual controls, was always present during the test.

Previous studies have demonstrated that a mean SDLP increase of 2.4 cm correlates with a BAC of 0.05% (Louwerens et al., 1987). This value was used as the prespecified non-inferiority margin for clinically relevant driving impairment in this study.

#### Subjective assessments of sedation, driving quality and mental effort to perform the driving test

Immediately before and after each driving test, each patient completed the Karolinska Sleepiness Scale (KSS) and reported their sleepiness on a scale ranging from 1 (‘extremely alert’) to 9 (‘very sleepy’, ‘great effort to keep awake’ and ‘fighting sleep’; Åkerstedt and Gillberg, 1990). Perceived driving quality was measured immediately after each driving test using a visual analogue scale from 0 (‘I drove exceptionally poorly’) to 20 (‘I drove exceptionally well’), with the midpoint indicating normal performance (‘I drove normally’). The level of mental effort needed to perform the driving test was assessed on a 15 cm visual analogue scale with markings ranging from ‘absolutely no effort’ to ‘extreme effort’ (Zijlstra and Van Doorn, 1985).

### Efficacy evaluations

#### Montgomery Åsberg Depression Rating Scale

Depression severity was assessed using the MADRS, a clinician-rated scale of depressive symptoms that is sensitive to changes due to antidepressant treatment (Montgomery and Asberg, 1979). Evaluated items on the MADRS are: ‘apparent sadness, reported sadness, inner tension, sleep, appetite, concentration, lassitude, inability to feel, pessimistic thoughts, and suicidal thoughts’. The 10 items can be scored from 0 (not present/normal) to 6 (severe or continuous presence of the symptoms; Montgomery and Asberg, 1979). MADRS was performed by trained physicians using a 7-day recall period or since last assessment. In Part A, the MADRS 7-day recall was performed at screening, prior to dosing on Day 1 for all treatment periods and at the follow-up visit, MADRS since last assessment was performed after completion of the driving assessment on Day 2 between 20 and 24 h after dosing. In Part B, MADRS 7-day recall was performed at screening, prior to dosing on Days 1, 11, 18 and 25, and at the follow-up visit.

### Safety assessments

In both parts of the study, safety assessments consisting of chemistry and haematology, urine drug screen, urinalysis, urine pregnancy tests for females, alcohol breath tests, electrocardiograms (ECGs), vital signs and a physical examination were performed prior to dosing of intranasal esketamine or intranasal placebo. Vital sign measurements were repeated 40 min after dosing. AEs were recorded throughout the study.

#### Columbia-Suicide Severity Rating Scale

Suicidal ideation and behaviour were assessed by the Columbia-Suicide Severity Rating Scale (C-SSRS), a clinician-rated questionnaire that is frequently used in clinical trials (Posner et al., 2011). Baseline C-SSRS was performed during screening to ascertain lifetime suicidal ideation and/or behaviour (Posner et al., 2011). Patients reporting suicidal ideations with intent and with or without specific plan within 6 months prior to screening or those reporting suicidal behaviour in the past year were excluded from the study. In Part A, the C-SSRS since last assessment was performed prior to dosing on Day 1 and the next day after the driving assessment. In Part B, the C-SSRS since last assessment was performed prior to and 2 h after dosing on Days 1, 11, 18 and 25.

#### Clinician-Administered Dissociative Symptoms Scale

Dissociative symptoms were measured by the Clinician-Administered Dissociative Symptoms Scale (CADSS), a clinician-administered questionnaire about depersonalization, derealization and amnesia (Bremner et al., 1998). In both study parts, the CADSS was performed prior to dosing at each visit, and at 1 and 2 h after dosing.

### Sample size

The sample size and power estimation were based upon the SDLP, the primary endpoint of the study. A non-inferiority margin of 2.4 cm in SDLP (associated with a BAC of 0.05%) that is considered clinically relevant was used for the power calculation (Verster and Roth, 2011). In line with a previous publication the true difference in SDLP between the active (esketamine) and placebo was assumed to be 0.63 cm (van de Loo et al., 2017). The intra-patient standard deviation (SD) for SDLP was assumed to be 2.1 cm based on previous literature (Verster and Roth, 2011). A conservative value for the SD of 2.97 cm for paired difference between active and placebo was used for the sample size calculation.

For Parts A and B with two-sided significance level of 0.05 (one-sided level of 0.025) for each comparison and an SD of 2.97 cm for paired differences, a sample size of 24 patients was assumed sufficient to achieve an 80% power.

The results of a planned interim analysis of driving performance data indicated that the within-patient variability of the SDLP was less than anticipated (1.43 cm vs expected 2.1 cm). It was therefore determined that the objectives of the study could be achieved with a sample size of 23 instead of 24 completers. Patients could be enrolled to participate in only Part B if Part A was already completed by sufficient patients.

### Statistical analysis

The statistical analyses were performed using SAS, version 9.4 (SAS Institute Inc., Cary, NC, USA). Mean and SD were computed for all outcome measures.

Part A: The statistical analysis of SDLP was conducted using a mixed effects model with treatment, sequence of treatments, and period as fixed effects and patients as a random effect. Within the model comparisons between active treatments and placebo (i.e. esketamine with alcohol placebo vs esketamine placebo with alcohol placebo and alcohol with esketamine placebo vs alcohol placebo with esketamine placebo) were conducted. The non-inferiority of esketamine compared to placebo was concluded if the upper limit of two-sided 95% confidence interval (CI) of the mean difference between the active (esketamine) and placebo was < 2.4 cm. Assay sensitivity was established if lower limit of two-sided 95% CI of mean difference between alcohol and placebo was > 0 cm.

Part B: The statistical analysis of SDLP was conducted using a mixed effects model with day of driving as a fixed effect and patient as a random effect. Pairwise comparisons between active treatments and placebo on each day of driving were conducted. For the SDLP, non-inferiority between treatments was concluded if the upper limit of two-sided 95% CI of the mean difference between the esketamine and esketamine placebo was < 2.4 cm.

Similar analyses (i.e. mixed effects model followed by calculation of two-sided 95% CIs of the mean difference between active and placebo) were performed for SDLP, SDS, MLP, MS, subjective driving assessments and KSS.

The MADRS total score and change from baseline were listed and summarized for each visit and timepoint.

## Results

### Demographics

In total, 27 patients, 18 females and nine males, with a mean (SD) age of 37.3 (10.6) years were enrolled in Part A of the study (Table 1). Mean (SD) total MADRS score at screening was 29.1 (5.1). Thirteen patients were receiving antidepressant treatment, the most common being sertraline (*n* = 3, dose range: 100–125 mg), citalopram (*n* = 3, dose range: 20–40 mg) and venlafaxine (*n* = 3, dose range: 75–150 mg).

**Table 1.** Demographics Parts A and B.

	Part A	Part B
	Total	Total
Patients enrolled	27	25
MADRS score screening		
Mean (SD)	29.1 (5.1)	NA
MADRS score baseline (pre-dose)		
Part A Placebo treatment Mean (SD)	23.1 (5.5)	NA
Part A Alcohol Treatment Mean (SD)	21.5 (6.49)	NA
Part A Esketamine treatment Mean (SD)	24.1 (4.5)	NA
Part B Day 1 Mean (SD)	NA	19.9 (6.6)
Age, years		
Mean (SD)	37.3 (10.6)	37.3 (10.6)
Sex		
Female	18 (66.7%)	17 (68.0%)
Males	9 (33.3%)	8 (32.0%)
Race		
White	24 (88.9%)	22 (88.0%)
Other	2 (7.4%)	2 (8.0%)
Multiple	1 (3.7%)	1 (4.0%)
Weight, kg		
Mean (SD)	70.2 (11.0)	71.6 (10.8)
Height, cm		
Mean (SD)	174.2 (8.6)	174.6 (8.8)
BMI, kg/m^2^		
Mean (SD)	23.3 (3.3)	23.4 (3.2)

MADRS: Montgomery Åsberg Depression Rating Scale; BMI: body mass index; NA: not applicable; SD: standard deviation.

In Part B, 25 patients were included, 17 females and eight males, with a mean (SD) age of 37.3 (10.6) years (Table 1). Mean (SD) total MADRS score at Day 1 of Part B was 19.9 (6.6). Concurrent antidepressant use was the same as in Part A.

### Withdrawals and missing data

**Figure 3.**
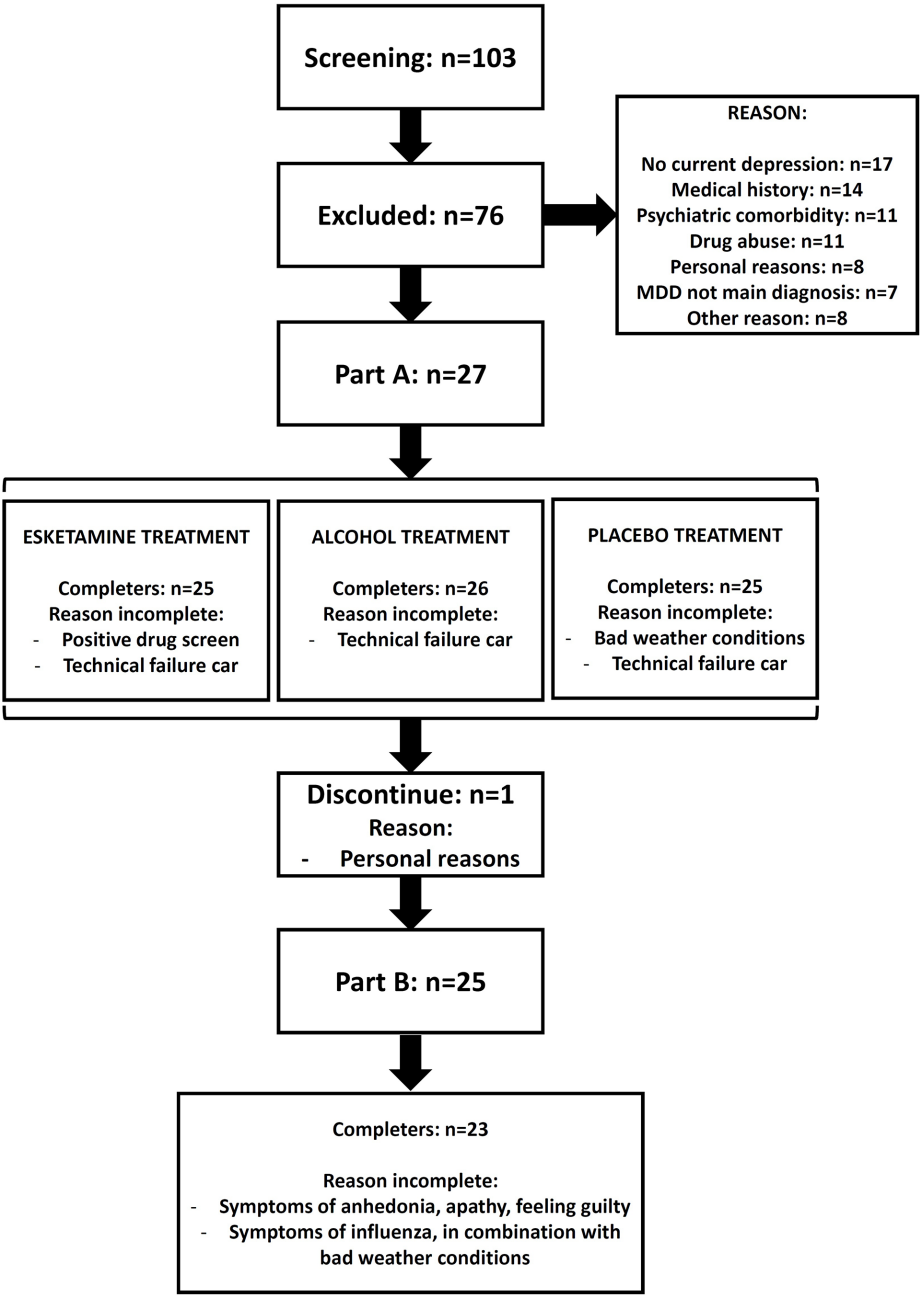
CONSORT diagram.

Part A was completed by 25 patients (eight men and 17 women; Figure 3). One patient could not start the last driving test due to weather conditions, but completed all safety assessments. Another patient was withdrawn from the study due to repeated positive urine screen for benzodiazepines. In one case, technical difficulties occurred with the test vehicle during the first visit of Part A in which the patient had received esketamine. Therefore, this patient did not complete the Part A visits and only participated in Part B. One patient discontinued for personal reasons after Part A. In total, 25 patients continued to participate in Part B of the study. In Part B, one patient with concomitant treatment of venlafaxine 112.5 mg, discontinued study participation after Day 8 due to symptoms of anhedonia, apathy and feeling guilty. These symptoms were considered possibly related to study drug by the investigator, and spontaneously resolved within 2 days after discontinuing the study. Another patient missed visit on Day 8 due to influenza (considered unrelated to study drug by the investigator) and missed visit on Day 25 due to a cancelled driving test because of extreme weather conditions. One patient in Part B did not perform the on-road driving test on Day 18 due to non-serious AEs of agitation, nausea, headache, somnolence and increased blood pressure considered mild in severity and possibly related to study drug by the investigator. This patient continued in the study, completing the final two dosing visits including the Day 25 driving test and was considered a completer of the study. Overall, 23 patients completed Part B of the study (Figure 3).

### Part A: Pharmacodynamic and pharmacokinetic results

#### Blood plasma concentrations esketamine

In Part A, the mean (SD) concentrations of esketamine and noresketamine in plasma collected at 1 h after dosing were 108 (31.1) and 92.6 (74.3) ng/mL, respectively.

#### Blood alcohol concentration

The mean (SD) BAC on Day 2 before the start of the driving test was 0.046% (0.003%), and after the driving test, it was 0.019% (0.005%).

#### Driving performance

The difference in least squares mean SDLP between esketamine and placebo was −0.23 cm, the upper limit of the two-sided 95% CI was 0.58 cm, which was below the non-inferiority criterion of 2.4 cm (Table 2). The difference in least squares mean SDLP between alcohol and placebo was + 1.83 cm. The lower limit of the two-sided 95% CI of the mean difference between alcohol and placebo was 1.03 cm, which met the criteria needed to demonstrate assay sensitivity (> 0 cm) (Table 2).

**Table 2.** Part A: Next-day on-road driving test results for single dose administration of esketamine (84 mg, intranasal), alcohol (BAC ⩽ 0.05%) and placebo.

*On-road driving test*	LSM			Difference of LSM, (95% CI), *p*-value	
	Placebo (*n* = 25)	Alcohol (*n* = 26)	Esketamine (*n* = 25)	Alcohol vs placebo	Esketamine vs placebo
SDLP (cm)	19.31	21.14	19.08	1.83 (1.03; 2.62) *p* < 0.001*	−0.23 (−1.04; 0.58) *p* = 0.572
SDS (km/h)	2.42	2.58	2.56	0.15 (−0.05; 0.36) *p* = 0.134	0.14 (−0.07; 0.35) p = 0.177
MLP (cm)	7.14	7.55	7.45	0.41 (−1.84; 2.65) *p* = 0.718	0.31 (−1.27; 2.58) *p* = 0.787
MS (km/h)	96.91	96.99	97.24	0.08 (−0.38; 0.55) *p* = 0.714	0.33 (−0.13; 0.80) *p* = 0.157
	LSM			Difference of LSM (95% CI), *p*-value	
*Subjective driving assessment*	Placebo (*n* = 25)	Alcohol (*n* = 26)	Esketamine (*n* = 25)	Alcohol vs placebo	Esketamine vs placebo
Perceived driving quality scale	9.53	8.92	9.74	−0.61 (−2.41; 1.18) *p* = 0.494	0.21 (−1.58; 1.99) *p* = 0.814
Perceived effort scale	6.87	6.74	6.67	−0.13 (−1.29; 1.03) *p* = 0.820	−0.20 (−1.31; 0.91) *p* = 0.716
*Subjective sleepiness assessment*	Placebo (*n* = 25)	Alcohol (*n* = 25)^a^	Esketamine (*n* = 25)	Alcohol vs placebo	Esketamine vs placebo
Pre-dose KSS scores	3.92	4.84	4.64	0.92 (0.31;1.53) *p* = 0.004*	0.72 (0.11;1.32) *p* = 0.021
Post-dose KSS scores	6.11	5.69	5.86	−0.42 (−1.13; 0.29) *p* = 0.241	−0.25 (−0.94; 0.44) *p* = 0.470

BAC: blood alcohol concentration; LSM: least squares mean; CI: confidence interval; SDLP: standard deviation of lateral position; SDS: standard deviation of speed; MLP: mean lateral position; MS: mean speed; KSS: Karolinska Sleepiness Scale.

a KSS by accident not performed in one patient.

Significant differences from placebo (*p*-values, two-sided, with a level of significance < 0.05) are indicated by ‘*’.

There were no statistically significant differences found for SDS, MLP or MS (Table 2).

#### Subjective assessments of sedation, driving quality and mental effort to perform the driving test

Prior to the driving test, KSS scores of both esketamine (least square mean estimates = 4.64, *p* = 0.021) and alcohol (least square mean estimates = 4.84, *p* = 0.004) were significantly higher than placebo (least square mean estimates = 3.92). After the driving tests, no significant difference in KSS scores were found after a dose of esketamine (least square mean estimates = 5.86, *p* = 0.241) or alcohol (least square mean estimates = 5.69, *p* = 0.470) compared to placebo (6.11).

No statistically significant differences between the treatments and placebo were found on subjective assessments of driving quality and mental effort to perform the driving tests (Table 2).

### Part B: Pharmacodynamic and pharmacokinetic results

#### Blood plasma concentrations of esketamine

In Part B, the mean (SD) concentrations of esketamine in plasma collected at 1 h post-dose on Days 11, 18 and 25 were 105 (26.0), 108 (31.0) and 104 (22.5) ng/mL, respectively. The mean (SD) noresketamine concentrations were 171 (95.7), 178 (83.4) and 183 (85.4) ng/mL, respectively.

#### Driving performance

On Day 11, the difference in least squares mean SDLP (cm) compared to Day 1 (upper limit of two-sided 95% CI (cm)) was −0.96 cm (1.81); Day 18, −0.56 cm (2.20) and Day 25, −1.05 cm (1.71), each below the non-inferiority criterion, 2.4 cm (Table 3).

**Table 3.** Part B: Same-day on-road driving test results for repeated dose administration of esketamine (84 mg, intranasal, Days 11, 18 and 25) compared to placebo (Day 1).

*On-road driving test*	LSM				Difference of LSM, (95% CI), *p*-value		
	Day 1 PCB (*n* = 25)	Day 11 (*n* = 23)	Day 18 (*n* = 23)	Day 25 (*n* = 23)	Days 11 vs 1	Days 18 vs 1	Days 25 vs 1
SDLP (cm)	18.72	17.76	18.15	17.66	−0.96 (−3.72; 1.81) p = 0.493	−0.56 (−3.33; 2.20) p = 0.686	−1.05 (−3.82; 1.71) p = 0.451
SDS (km/h)	2.73	2.66	2.50	2.30	−0.06 (−0.69; 0.57) p = 0.847	−0.22 (−0.85; 0.40) p = 0.479	−0.42 (−1.05; 0.20) p = 0.183
MLP (cm)	9.84	10.06	11.36	9.07	0.23 (−6.21; 6.66) p = 0.945	1.52 (−4.92; 7.96) p = 0.640	−0.76 (−7.20; 5.67) p = 0.814
MS (km/h)	96.91	97.52	97.41	97.43	0.61 (−0.48; 1.69) p = 0.268	0.50 (−0.59; 1.58) p = 0.364	0.52 (−0.57; 1.60) p = 0.344
*Subjective driving assessment*	Day 1 PCB (*n* = 25)	Day 11 (*n* = 23)	Day 18 (*n* = 23)	Day 25 (*n* = 23)	Days 11 vs 1	Days 18 vs 1	Days 25 vs 1
Perceived driving quality scale	10.83	10.90	11.13	11.63	0.07 (−2.05; 2.19) *p* = 0.949	0.30 (−1.82; 2.42) *p* = 0.781	0.80 (−1.35; 2.94) *p* = 0.464
Perceived effort scale	5.25	5.67	5.22	5.11	0.42 (−1.14; 1.98) *p* = 0.592	−0.03 (−1.57; 1.52) *p* = 0.971	−0.13 (−1.68; 1.41) *p* = 0.865
*Subjective sleepiness assessment*	Day 1 PCB (*n* = 25)	Day 11 (*n* = 23)	Day 18 (*n* = 23)	Day 25 (*n* = 23)	Days 11 vs 1	Days 18 vs 1	Days 25 vs 1
Pre-dose KSS scores	4.20	4.78	4.52	4.83	0.58 (−0.49; 1.66) *p* = 0.285	0.32 (−0.76; 1.40) *p* = 0.554	0.63 (−0.45; 1.70) *p* = 0.251
Post-dose KSS scores	5.0	5.2	5.3	5.2	0.17 (−0.97; 1.32) *p* = 0.763	0.30 (−0.89; 1.49) *p* = 0.617	0.18 (−0.97; 1.34) *p* = 0.755

On Day 1, placebo nasal spray dosing; on remaining days, 84 mg esketamine nasal spray dosing.

LSM: least squares mean; CI: confidence interval; PCB: placebo; SDLP: standard deviation of lateral position; SDS: standard deviation of speed; MLP: mean lateral position; MS: mean speed; KSS: Karolinska Sleepiness Scale.

None of the outcome measures after esketamine differed significantly from placebo (two-sided, *p* < 0.05).

There were no statistically significant differences observed between esketamine and placebo for SDS, MLP and MS (Table 3).

#### Subjective assessment of sedation, driving quality and mental effort to perform the driving test

On KSS scores prior to the driving test, no statistically significant difference was observed between placebo treatment Day 1 (least square mean estimates = 4.20) and intranasal esketamine treatment on Day 11 (least square mean estimates = 4.78, *p* = 0.285), Day 18 (least square mean estimates = 4.52, *p* = 0.554) or Day 25 (least square mean estimates = 4.83, *p* = 0.251), respectively. For assessments made after completion of the driving tests, no statistically significant differences were observed. No significant differences between the esketamine and placebo were found on subjective assessments of driving quality and mental effort to perform the driving tests.

### Efficacy and safety results Part A and Part B

#### MADRS

**Figure 4.**
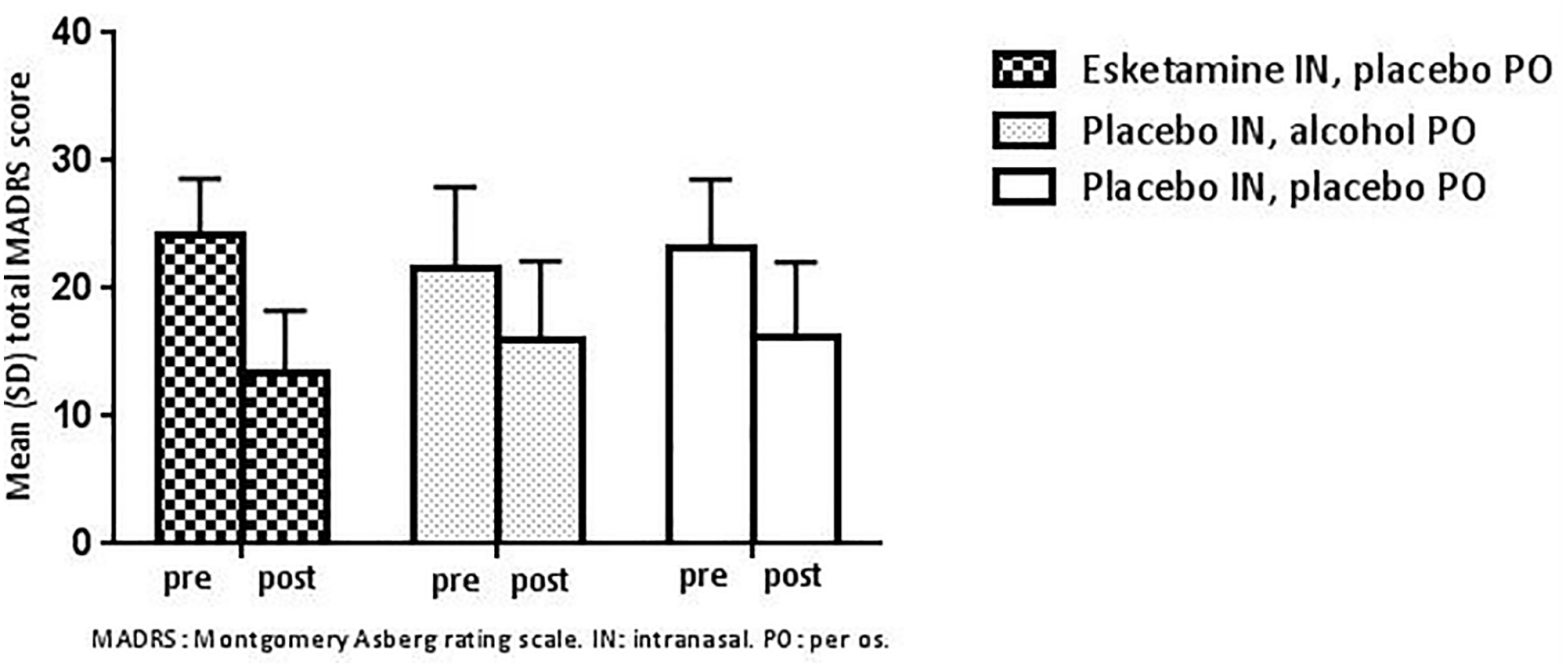
Mean (SD) total MADRS score before (pre) and 20–24 h after (post) administration of single-dose intranasal placebo with oral placebo or oral alcohol, or single-dose 84 mg intranasal esketamine with oral placebo (Part A).

At screening, the mean (SD) MADRS total score was 29.1 (5.07). On Day 1 (prior to dosing, respective treatment periods), the mean (SD) MADRS total score for Part A were comparable across treatment periods, 24.1 (4.51), 23.1 (5.48) and 21.5 (6.49) for intranasal esketamine + oral placebo, intranasal placebo + oral placebo and intranasal placebo + oral alcohol, respectively. The mean (SD) MADRS total score changes from baseline were: −10.7 (4.11), −6.8 (5.29) and −5.5 (6.88) for intranasal esketamine + oral placebo, intranasal placebo + oral placebo and intranasal placebo + oral alcohol, respectively (Figure 4).

**Figure 5.**
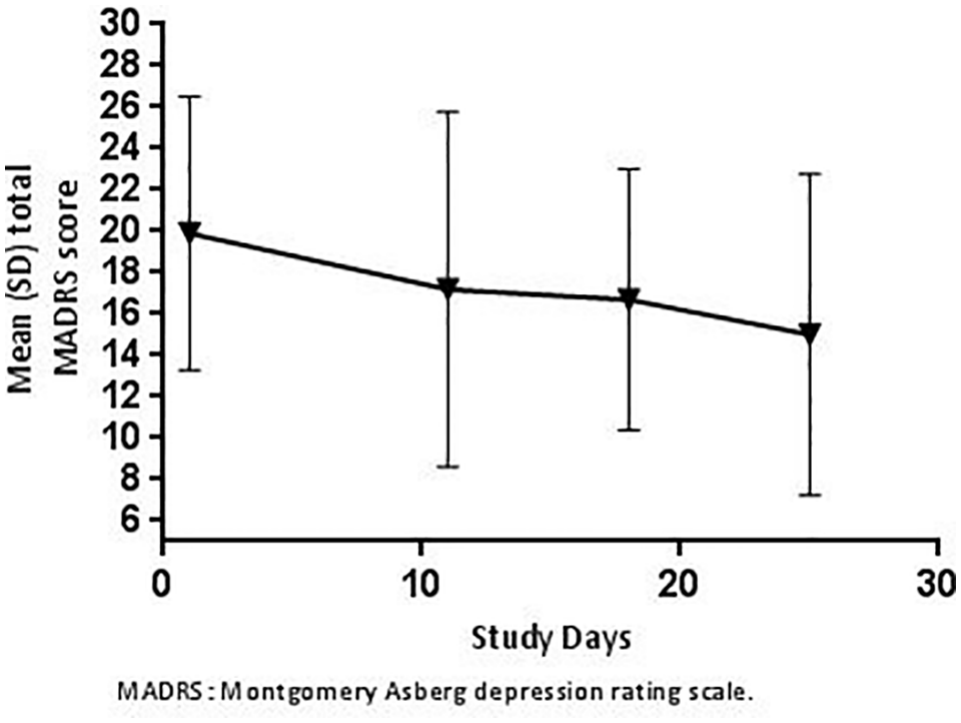
Mean (SD) total MADRS score prior to each dose of 84 mg intranasal esketamine on subsequent study visits (Part B).

During Part B of the study, the mean (SD) MADRS total score at baseline (Day 1, prior to dosing) was 19.9 (6.61). After repeated administration of intranasal esketamine, the mean (SD) changes in MADRS total score were −2.3 (6.41), −3.0 (6.04) and −5.2 (8.36) on Days 11, 18 and 25, respectively (Figure 5).

At the follow-up visit, which occurred after completing Parts A and B, or if the patient early withdrew, the mean (SD) MADRS total score was 16.5 (8.64).

#### C-SSRS

At screening, 11 patients reported to have had suicidal ideations with intent/no plan during lifetime, and three patients reported suicidal ideations with plan/intent during lifetime. After the screening phase, none of the patients that received esketamine reported suicidal ideation with intent/no plan or suicidal ideations with plan/intent.

#### CADSS

During Part A of the study, the mean (SD) CADSS total score increased to 11.6 (7.84) 1 h following esketamine, and subsequently decreased to a mean score of 1.3 (2.57) at 2 h post-dose.

During Part B of the study, the mean (SD) CADSS total scores 1 h following esketamine increased to 6.3 (8.03), 5.0 (9.28) and 5.0 (8.22), and decreased to 0.3 (0.88), 0.5 (1.87) and 0.4 (1.50) at 2 h after dosing, on Days 11, 18 and 25, respectively.

#### Adverse events

No serious AEs or deaths were reported in the study. In Part A, 77.8% of treatment-emergent adverse events (TEAEs) were assessed as mild, the remaining 22.2% were moderate. In Part B, 76.0% of TEAEs were assessed as mild and 24.0% as moderate. Across the study, most AEs resolved within 2 h and none of the AEs restricted patient discharge. The most commonly reported AEs in Part A were (reported by ⩾ 30% of patients): intranasal esketamine: dissociation (80.8%), dizziness (69.2%), paraesthesia (46.2%) and paraesthesia oral (38.5%); oral alcohol: headache (34.6%) and dizziness (30.8 %). For intranasal placebo: no AEs meeting the 30% threshold were reported.

Cumulative AEs commonly reported in Part B were: dissociation (96.0%), dizziness (84.0%), dysgeusia (72.0%), fatigue (56.0%), headache (56.0%), paraesthesia oral (52.0%), paraesthesia (52.0%), somnolence (48.0%), nasal discomfort (48.0%), mild blood pressure increased (36.0%) and vision blurred (32.0%).

One patient reported suicidal ideation with plan/intent during the end of study visit. The patient was discontinued during Period 3 of Part A due to repeated positive tests for benzodiazepines and was never dosed with esketamine.

Another patient reported symptoms of epistaxis, nasal discomfort and nasopharyngitis on Day 1 of Part B (placebo treatment) and completed the visits. During the follow-up visit, an irritated nasal mucosa and bilateral nasal septum lesions (ca. 0.5 cm) were observed. The patient was diagnosed with a nasal septum perforation by an ear nose throat specialist and treated with a nasal septum button. This AE was considered mild and possibly related to the study drug by the investigator.

No clinically meaningful changes in haematology, biochemistry and urinalysis parameters were observed. On ECG, no clinically meaningful treatment-related changes were observed.

## Discussion

In this study, the effects of intranasally administered esketamine on driving performance were assessed in unipolar depressed MDD or PDD patients. The design of this study included many of the components that are recommended to be included in an assessment of the effects of a drug on driving performance (FDA, 2017). For example, during this study, subjects received a placebo (Parts A and B) and positive control (Part A only), the latter to confirm assay sensitivity. Driving was assessed to evaluate both the effects after initial drug exposure and after chronic exposure at the highest clinically relevant dose of intranasal esketamine. Furthermore, subjects with MDD were eligible for enrolment, which were expected to be more comparable to the population for which intranasal esketamine is intended (i.e. subjects with TRD) relative to healthy subjects.

Assay sensitivity was confirmed by significant impairment on SDLP driving performance with alcohol. In the studied patient population, next-day driving performance assessed approximately 18 h after dosing, including a night of sleep, was not impaired by a single intranasal dose of 84 mg esketamine. Similarly, twice weekly doses of 84 mg intranasal esketamine did not affect same-day driving performance assessed approximately 6 h after dosing. While one could argue that the lack of an effect of intranasal esketamine on driving performance in the studied population might be due to symptom relief, we consider this as unlikely as in a previous healthy volunteer study, no effects a single intranasal dose of 84 mg esketamine on driving performance were observed at 8 h after dosing (van de Loo et al., 2017).

In Part A, subjective sleepiness was statistically significantly higher for both esketamine and alcohol compared with placebo 18 h post-dosing and immediately prior to the driving test. These effects were considered not clinically relevant because mean scores fell between the scale anchors ‘alert’ and ‘neither alert, nor sleepy’, and therefore were not expected to influence driving performance. For both treatments (esketamine and alcohol) the KSS scores following completion of the driving assessment were not different relative to placebo. In contrast, following repeated administration of esketamine in Part B, subjective sleepiness was stable on all days tested. Subjective assessments of driving quality and mental effort to perform the driving tests remained unaffected in both next- and same-day driving following repeat dosing.

This study was not designed or powered to measure efficacy of intranasal esketamine for the treatment of depressive symptoms in MDD or PDD patients, but MADRS scores were collected during the study as an exploratory, secondary endpoint. In both Parts A and B of the study, a trend towards a decrease in depressive symptoms was observed, however, this was not tested for statistical significance. Although smaller than the decrease after esketamine treatment, a trend towards decrease of depressive symptoms was also observed after placebo treatment in Part A. This relatively substantial placebo effect might have been influenced by factors such as patients having awareness of study participation, attention and care by research staff and normal fluctuation of symptom severity over time. It is important to note that such placebo effect is rather common in depression trials, which puts these findings in perspective (Sonawalla and Rosenbaum, 2002). Together, we believe that these exploratory efficacy results in MDD or PDD patients are informative but need to be interpreted with caution.

Several limitations of the present study are worth mentioning. Limitations include the risk of treatment bias due to potential unblinding in Part A of the study and the open-label design of the same-day driving assessment (Part B). This open-label design was chosen for practical reasons, but also reflects the real-life situation as patients undergoing esketamine treatment are aware of this. The next-day driving assessment was single blind to ensure adherence to local laws and restrictions in terms of allowed BAC when driving. The study aimed to minimize the risk of treatment bias in the single-dose part of the study by also conducting BAC measurements in the placebo condition and instructing the research staff not to inform patients about the treatment being administered. However, patient unblinding cannot unequivocally be ruled out as many people are familiar with the effects of consuming alcohol.

In line with other studies, intranasal esketamine was generally well tolerated and no drug-related serious adverse events (SAEs) were observed (Daly et al., 2018, 2019; Popova et al., 2019; Wajs et al., 2020). Furthermore, most AEs resolved spontaneously within 2 h. Consistent with earlier observations, dissociative symptoms as measured by the CADSS generally resolved within 2 h (Daly et al., 2018, 2019; Popova et al., 2019). In addition, consistent with other efficacy studies, no clinically relevant effect on suicidal ideations after esketamine treatment was observed (Daly et al., 2018, 2019; Popova et al., 2019).

To conclude, in unipolar depressed MDD or PDD patients, 84 mg intranasal esketamine, the highest recommended dose in prescribing information in the United States and other countries worldwide including those in Europe did not impair on-road driving performance, the next day after a single administration and a night of sleep, or 6 h after repeated dosing over a period of 3 weeks.

## References

[bibr1-02698811221078764] Åkerstedt T Gillberg M (1990) Subjective and objective sleepiness in the active individual. International Journal of Neuroscience 52: 29–37. 2265922 10.3109/00207459008994241

[bibr2-02698811221078764] Bremner JD Krystal JH Putnam FW , et al. (1998) Measurement of dissociative states with the Clinician-Administered Dissociative States Scale (CADSS). Journal of Traumatic Stress 11: 125–136. 9479681 10.1023/A:1024465317902

[bibr3-02698811221078764] Daly EJ Singh JB Fedgchin M , et al. (2018) Efficacy and safety of intranasal esketamine adjunctive to oral antidepressant therapy in treatment-resistant depression: A randomized clinical trial. JAMA Psychiatry 75: 139–148. 29282469 10.1001/jamapsychiatry.2017.3739 PMC5838571

[bibr4-02698811221078764] Daly EJ Trivedi MH Janik A , et al. (2019) Efficacy of esketamine nasal spray plus oral antidepressant treatment for relapse prevention in patients with treatment-resistant depression: A randomized clinical trial. JAMA Psychiatry 76: 893–903. 31166571 10.1001/jamapsychiatry.2019.1189 PMC6551577

[bibr5-02698811221078764] Fava M (2003) Diagnosis and definition of treatment-resistant depression. Biological Psychiatry 53: 649–659. 12706951 10.1016/s0006-3223(03)00231-2

[bibr6-02698811221078764] Friel PN Logan BK O’Malley D , et al. (1999) Development of dosing guidelines for reaching selected target breath alcohol concentrations. Journal of Studies on Alcohol and Drugs 60: 555–565. 10.15288/jsa.1999.60.555 10463813

[bibr7-02698811221078764] James SL Abate D Abate KH , et al. (2018) Global, regional, and national incidence, prevalence, and years lived with disability for 354 diseases and injuries for 195 countries and territories, 1990-2017: A systematic analysis for the Global Burden of Disease Study 2017. The Lancet 392: 1789–1858. 10.1016/S0140-6736(18)32279-7 PMC6227754 30496104

[bibr8-02698811221078764] Louwerens JW Gloerich ABM De Vries G , et al. (1987) The relationship between drivers’ blood alcohol concentration and actual driving performance. In: Noordzij PC Roszbach R (eds) Alcohol, Drugs and Traffic Safety-T86. Amsterdam: Excerpta Medica, pp.183–192.

[bibr9-02698811221078764] Montgomery SA Asberg M (1979) A new depression scale designed to be sensitive to change. British Journal of Psychiatry 134: 382–389. 10.1192/bjp.134.4.382 444788

[bibr10-02698811221078764] Murrough JW Charney DS (2012) Is there anything really novel on the antidepressant horizon? Current Psychiatry Reports 14: 643–649. 22996298 10.1007/s11920-012-0321-8 PMC3662536

[bibr11-02698811221078764] O’Hanlon JF Haak TW Blaauw GJ , et al. (1982) Diazepam impairs lateral position control in highway driving. Science 217: 79–81. 7089544 10.1126/science.7089544

[bibr12-02698811221078764] Popova V Daly EJ Trivedi M , et al. (2019) Efficacy and safety of flexibly dosed esketamine nasal spray combined with a newly initiated oral antidepressant in treatment-resistant depression: A randomized double-blind active-controlled study. The American Journal of Psychiatry 176: 428–438. 31109201 10.1176/appi.ajp.2019.19020172

[bibr13-02698811221078764] Posner K Brown GK Stanley B , et al. (2011) The Columbia-Suicide Severity Rating Scale: Initial validity and internal consistency findings from three multisite studies with adolescents and adults. The American Journal of Psychiatry 168: 1266–1277. 22193671 10.1176/appi.ajp.2011.10111704 PMC3893686

[bibr14-02698811221078764] Singh JB Fedgchin M Daly E , et al. (2016) Intravenous esketamine in adult treatment-resistant depression: A double-blind, double-randomization, placebo-controlled study. Biological Psychiatry 80: 424–431. 26707087 10.1016/j.biopsych.2015.10.018

[bibr15-02698811221078764] Sonawalla SB Rosenbaum JF (2002) Placebo response in depression. Dialogues in Clinical Neuroscience 4: 105–113. Available at: https://www.ncbi.nlm.nih.gov/pmc/articles/PMC3181672/22034204 10.31887/DCNS.2002.4.1/ssonawalla PMC3181672

[bibr16-02698811221078764] SPRAVATO (2020) SPRAVATO (Esketamine) Nasal Spray [Prescribing Information]. Titusville, NJ: Janssen Pharmaceuticals.

[bibr17-02698811221078764] SPRAVATO (esketamine) nasal spray EMA summary of product characteristics. Available at: https://www.ema.europa.eu/en/documents/product-information/spravato-epar-product-information_en.pdf

[bibr18-02698811221078764] U.S. Food and Drug Administration (2017) Evaluating drug effects on the ability to operate a motor vehicle (Guidance for industry). Available at: https://www.fda.gov/Drugs/GuidanceComplianceRegulatoryInformation/Guidances/default.htm

[bibr19-02698811221078764] Vaa T (2003) Impairments, diseases, age, and their relative risks of traffic accident involvement: Results from meta-analysis. Report 690/2003. Oslo, Norway: The Institute of Transport Economics.

[bibr20-02698811221078764] van de Loo A Bervoets AC Mooren L , et al. (2017) The effects of intranasal esketamine (84 mg) and oral mirtazapine (30 mg) on on-road driving performance: A double-blind, placebo-controlled study. Psychopharmacology 234: 3175–3183. 28755104 10.1007/s00213-017-4706-6 PMC5660834

[bibr21-02698811221078764] Verster JC Mets MAJ (2009) Psychoactive medication and traffic safety. International Journal of Environmental Research and Public Health 6: 1041–1054. 19440432 10.3390/ijerph6031041 PMC2672393

[bibr22-02698811221078764] Verster JC Roth T (2011) Standard operation procedures for conducting the on-the-road driving test, and measurement of the standard deviation of lateral position (SDLP). International Journal of General Medicine 4: 359–371. 21625472 10.2147/IJGM.S19639 PMC3100218

[bibr23-02698811221078764] Vos T Flaxman AD Naghavi M , et al. (2012) Years lived with disability (YLDs) for 1160 sequelae of 289 diseases and injuries 1990-2010: A systematic analysis for the Global Burden of Disease Study 2010. The Lancet 380: 2163–2196. 10.1016/S0140-6736(12)61729-2 PMC6350784 23245607

[bibr24-02698811221078764] Wajs E Aluisio L Holder R , et al. (2020) Esketamine nasal spray plus oral antidepressant in patients with treatment-resistant depression: Assessment of long-term safety in a phase 3, open-label study (SUSTAIN-2). The Journal of Clinical Psychiatry 81(3): 19m12891. 10.4088/JCP.19m12891 32316080

[bibr25-02698811221078764] Walker ER McGee RE Druss BG (2015) Mortality in mental disorders and global disease burden implications: A systematic review and meta-analysis. JAMA Psychiatry 72: 334–341. 25671328 10.1001/jamapsychiatry.2014.2502 PMC4461039

[bibr26-02698811221078764] Zijlstra FRH Van Doorn L (1985) The Construction of a Scale to Measure Perceived Effort. Delft: Delft University of Technology.

